# Liverpool Sanatorium for Consumptives

**Published:** 1902-09-20

**Authors:** 


### LIVERPOOL SANATORIUM FOR CONSUMPTIVES.

From Liverpool the traveller leaves the train at Frodsham, and from this old-fashioned Cheshire village a drive of three and a half miles up the brow of the hillside brings him to Kingswood, where, on a substantial plot of 40 acres, stands the new Sanatorium for Liverpool—an institution which may be looked on as pioneer in the treatment of consumption occurring in that class of patients which will not accept gratuitous medical aid and cannot pay the charges in sanatoria for the well-to-do classes.

The site lies on the outskirts of Delamere Forest, and the building occupies an almost ideal position. It is sufficiently removed from every centre of population to ensure that the air shall at all times be pure, and being 500 feet above the sea-level the air will always be in motion. As the site is quite open to the south it will be understood by everyone that the view is extensive, and we may state that it is as pleasing as it is extensive. About eight miles to the south-west the old city of Chester can be made out, when not hidden in mist or in its own smoke, and to the west lies the Mersey, with part of the Ship Canal in sight.

The Sanatorium at present contains about forty patients, and the class from which they are drawn may be described as that of the lower middle class. The charges for residence and treatment are very moderate, namely, £1 a week, and a few cases are admitted as low as 12s. 6d. if they receive subscriber's ticket. The example set by Liverpool is one which we hope to see very extensively followed, for it is exactly this class on which sickness has hitherto pressed most heavily. We might almost say that hospitals and asylums have been provided in profusion for the poor, and there are plenty of homes for the rich, but for those able to pay from 10s. to £1 a week the accommodation is hopelessly insufficient. The more credit, therefore, to the Liverpool people who have seen the blot and have helped to remove it as far as consumption is concerned.

The building, that is the patients' part of it, is linear in design, of two stories in height, and, of course, it faces the south. On this aspect are the verandahs, and a corridor runs along the north side, providing communication with the patients' rooms and with the corridor which connects the dining-hall and administration part. The rooms vary in size, but perhaps something like 15 feet by 12 feet may be taken as an average. The windows are large and take up the greater part of the south elevation. Cross ventilation is obtained by keeping the doors of the rooms open. The woodwork seems to have been very carefully done.

The sanitary annexes are placed at the ends. They are lined with glazed bricks, and the baths are nicely fitted up. We should like to have seen a little more space in some of the subdivisions of these annexes. The administrative department is correctly placed to the north. It contains a good dining-room and a fair-sized kitchen, the former being conveniently placed in relation to the patients' rooms, and the latter in close juxtaposition to the dining room and having its offices grouped around it.

The main entrance to the Sanatorium is in the south front. This we look upon as a mistake, because patients should be as little overlooked as possible by strangers visiting the Sanatorium for any purpose. Again, we should have placed the sewage-settling pits at least twice as far from the building as they are, but we should add that there was no smell from them on the occasion of our visit. The water is obtained from the public waterworks.

We fear it will soon be proved the Sanatorium is not nearly large enough for such a populous city as Liverpool, and considerable additions will have to be made to it before long if it is to extend its usefulness. At present more bungalows are being built under the eye of the resident medical officer, and these are a distinct advance on the others which were designed before that gentleman's arrival. We think the committee have been exceptionally fortunate in obtaining the services of Dr. Wood, and we look for many improvements and suggestions from him in the construction of these buildings which are as yet in their infancy among us.

In a building so well adapted for its purpose as this is we may look for a high percentage of cures and improvements. As yet it has not been open long enough to produce statistics sufficiently extensive to found any reliable data on; but the institution will doubtless publish an annual report, and we hope to have the pleasure of noticing it in some future number.

